# Integrating Redox Proteomics and Computational Modeling to Decipher Thiol-Based Oxidative Post-Translational Modifications (oxiPTMs) in Plant Stress Physiology

**DOI:** 10.3390/ijms26146925

**Published:** 2025-07-18

**Authors:** Cengiz Kaya, Francisco J. Corpas

**Affiliations:** 1 Soil Science and Plant Nutrition Department, Harran University, Sanliurfa 63200, Turkey; c_kaya70@yahoo.com; 2 Group of Antioxidants, Free Radicals and Nitric Oxide in Biotechnology, Food and Agriculture, Department of Stress, Development and Signaling in Plants, Estación Experimental del Zaidín, Spanish National Research Council (CSIC), C/Profesor Albareda 1, 18008 Granada, Spain

**Keywords:** nitric oxide, redox proteomics, thiol-based oxidative post-translational modifications (oxiPTMs), computational modeling, machine learning, redox signaling networks

## Abstract

Redox signaling is central to plant adaptation, influencing metabolic regulation, stress responses, and developmental processes through thiol-based oxidative post-translational modifications (oxiPTMs) of redox-sensitive proteins. These modifications, particularly those involving cysteine (Cys) residues, act as molecular switches that alter protein function, structure, and interactions. Advances in mass spectrometry-based redox proteomics have greatly enhanced the identification and quantification of oxiPTMs, enabling a more refined understanding of redox dynamics in plant cells. In parallel, the emergence of computational modeling, artificial intelligence (AI), and machine learning (ML) has revolutionized the ability to predict redox-sensitive residues and characterize redox-dependent signaling networks. This review provides a comprehensive synthesis of methodological advancements in redox proteomics, including enrichment strategies, quantification techniques, and real-time redox sensing technologies. It also explores the integration of computational tools for predicting *S*-nitrosation, sulfenylation, *S*-glutathionylation, persulfidation, and disulfide bond formation, highlighting key models such as CysQuant, BiGRUD-SA, DLF-Sul, and Plant PTM Viewer. Furthermore, the functional significance of redox modifications is examined in plant development, seed germination, fruit ripening, and pathogen responses. By bridging experimental proteomics with AI-driven prediction platforms, this review underscores the future potential of integrated redox systems biology and emphasizes the importance of validating computational predictions, through experimental proteomics, for enhancing crop resilience, metabolic efficiency, and precision agriculture under climate variability.

## 1. Introduction

Plants continuously encounter environmental challenges such as drought, salinity, and pathogen attacks, requiring highly regulated adaptive mechanisms. Among these, redox signaling plays a fundamental role in modulating plant responses by mediating reversible thiol-based oxidative post-translational modifications (oxiPTMs) of proteins. Redox-sensitive proteins, particularly those containing reactive cysteine (Cys) residues, act as molecular switches that regulate cellular processes through modifications such as *S*-nitrosation, sulfenylation, *S*-glutathionylation, persulfidation, and disulfide bond formation [1,2,3,4]. These modifications not only alter protein structure, stability, and function but also serve as key regulatory mechanisms in plant stress responses and metabolic adjustments.

Understanding redox-sensitive proteins and their functional significance has remained a substantial challenge due to the dynamic and reversible nature of oxidative modifications. Traditional biochemical methods often lack the sensitivity and specificity required to detect transient oxidative modifications in a physiological context. The advent of redox proteomics, leveraging advanced mass spectrometry-based techniques, has provided unprecedented insights into the functional dynamics of redox-sensitive proteins [5,6]. These advancements have facilitated the identification of previously undetected oxiPTMs, broadening our understanding of redox-regulated cellular processes.

Redox proteomics has been instrumental in discovering numerous redox-sensitive proteins involved in key cellular processes, including photosynthesis, guard cell signaling, fruit ripening, and stress adaptation [7,8]. Enabling the characterization of oxidative modifications has deepened our knowledge of how redox regulation influences plant growth, development, and environmental interactions. In recent years, the integration of redox proteomics with multi-omics approaches, such as transcriptomics, metabolomics, and lipidomics, has provided a holistic view of redox regulatory networks [9,10,11,12]. These integrative strategies help uncover cross-talk between different signaling pathways, allowing a systems-level understanding of redox-dependent metabolic reprogramming and plant stress responses [1,13]. Furthermore, integrating redox proteomics with systems biology, computational modeling, and metabolomics approaches has provided a holistic view of redox regulatory networks, opening new avenues for plant stress resilience research [14,15].

Recent breakthroughs in computational biology, artificial intelligence (AI), and machine learning (ML) have further expanded the scope of redox proteomics. AI-driven predictive models and deep learning algorithms are now capable of identifying potential redox-sensitive sites, predicting oxidative modifications, and uncovering novel regulatory mechanisms with high precision [16,17]. Tools such as CysQuant [18], BiGRUD-SA [19], DLF-Sul [20], and iCarPS [21] utilize machine learning frameworks to refine redox PTM predictions, enabling large-scale functional annotation of redox-modified proteins. These computational approaches are transforming redox biology from a largely descriptive field into one that can predict and manipulate redox-dependent processes, offering exciting possibilities for precision agriculture and stress-resilient crop development [22,23].

Despite advancements in redox proteomics, most previous reviews have primarily focused on either mass spectrometry-based profiling of redox PTMs [24] or the theoretical landscape of Cys redox proteoforms [25]. However, there remains a critical gap in understanding how computational modeling can enhance redox proteomics for functional characterization and predictive analysis. This review aims to provide a comprehensive overview of reversible thiol-based redox post-translational modifications (oxiPTMs) that target Cys residues in plants. These modifications, including *S*-sulfenylation, *S*-nitrosation, *S*-glutathionylation, and persulfidation, function as redox switches that dynamically regulate protein function, signaling pathways, and plant adaptation to environmental stress. Emphasis is placed on their biochemical mechanisms, roles in cellular signaling networks, and emerging computational tools for their prediction. By focusing specifically on Cys-centered redox regulation, this review highlights the integrative role of these modifications in plant stress responses, development, and redox homeostasis.

## 2. Redox Proteomics in Plant Biology

**Figure 1:**
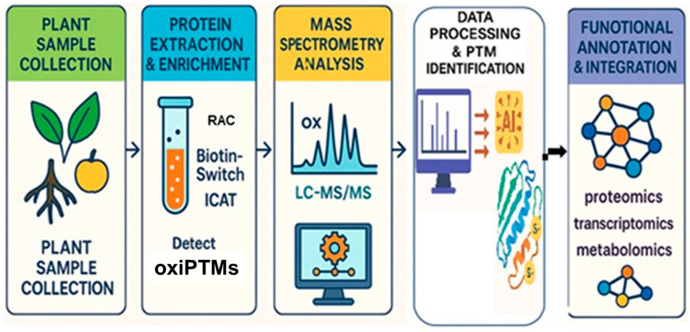
Overview of redox proteomics workflow—this figure illustrates the key steps involved in redox proteomics, from sample collection to data integration. Plant sample collection—representative plant tissues, including leaves, roots, and fruits, are collected for protein extraction. Protein extraction and enrichment—proteins are extracted and enriched using specialized techniques such as Resin-Assisted Capture (RAC), Biotin-Switch Assay, and Isotope-Coded Affinity Tags (ICATs). Mass spectrometry analysis—high-resolution mass spectrometry (LC-MS/MS) is used to detect oxidative post-translational modifications (oxiPTMs). Data processing and PTM identification—bioinformatics tools and AI-based algorithms analyze mass spectrometry data to identify and characterize redox-sensitive proteins. Functional annotation and integration—identified redox modifications are integrated with multi-omics approaches (proteomics, transcriptomics, and metabolomics) to map regulatory networks and understand stress resilience in plants.

Redox proteomics, a specialized branch of proteomics, has revolutionized the study of oxiPTMs by enabling precise detection, quantification, and functional annotation of redox-sensitive proteins in a physiological context [5,6,26]. These proteins, typically modified at reactive Cys residues, serve as molecular switches that regulate activity, stability, and interactions in response to cellular redox changes [7]. Their roles span signaling, metabolism, stress responses, and redox homeostasis, and are central to plant adaptation under environmental challenges [1]. OxiPTMs such as *S*-glutathionylation, *S*-nitrosation, persulfidation, disulfide bond formation, and sulfenylation have been characterized. However, due to their transient nature and relatively low abundance, these modifications are difficult to detect with conventional biochemical methods [27]. Modern redox proteomics overcomes these challenges through integrated workflows involving sample collection, protein extraction and enrichment, mass spectrometry (LC-MS/MS), and data analysis (Figure 1).

### 2.1. Methodological Advances in Redox Proteomics

Mass spectrometry (MS)-based redox proteomics has enabled high-throughput profiling of redox-sensitive proteins and oxiPTMs with greater precision. To increase specificity and sensitivity, enrichment methods such as Isotope-Coded Affinity Tags (ICATs), Resin-Assisted Capture (RAC), and the Biotin-Switch Assay have been developed. ICATs allow for quantification of oxidized versus reduced cysteines using isotopically labeled tags [28]. RAC selectively captures thiol-containing peptides, enhancing detection of redox modifications [29]. The Biotin-Switch Assay is particularly effective for detecting *S*-nitrosation, a key NO-mediated modification, by converting modified Cys to biotin-tagged forms [30]. Quantitative labeling strategies such as OxICAT and iodoTMT offer site-specific quantification and enable differentiation between regulatory and stress-induced modifications [24,31]. Collectively, these techniques have significantly advanced the reliability and depth of redox proteomic analyses.

### 2.2. Redox-Driven Signaling Pathways and Stress Responses

**Figure 2:**
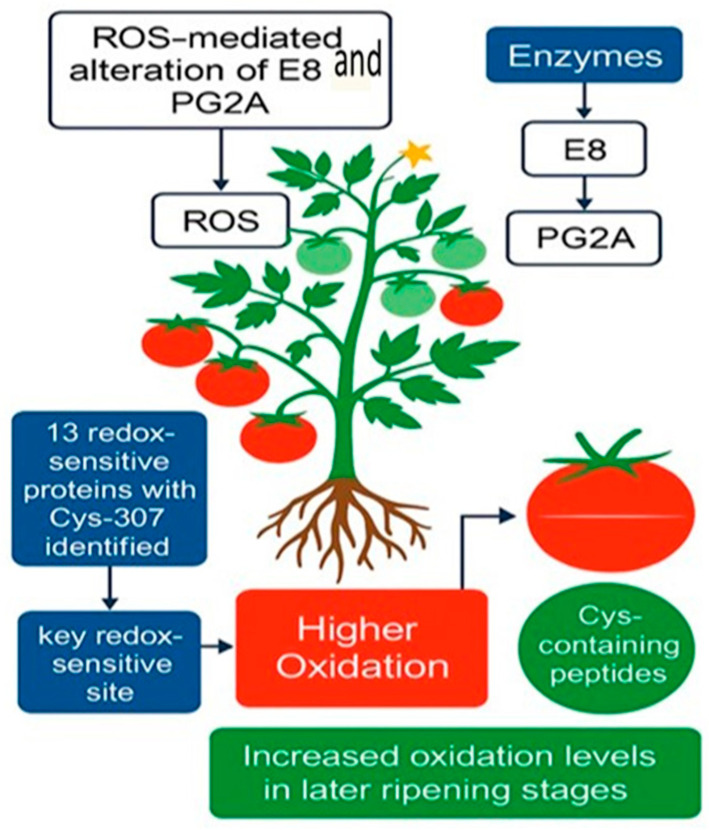
A proposed model of ROS-mediated regulation in tomato fruit ripening, based on iodoTMT (iodoacetyl tandem mass tag-labeled) redox proteomics, reveals that reactive oxygen species (ROS) dynamically modulate critical enzymes such as polygalacturonase 2A (PG2A) and 1-aminocyclopropane-1-carboxylate oxidase-like protein (E8). The investigation identified 13 redox-sensitive proteins, with Cys-307 in E8 pinpointed as a key redox-sensitive site. Higher oxidation levels observed in Cys-containing peptides during later ripening stages suggest a regulatory role for ROS in fruit ripening [35]. Cys, cysteine.2.3. Enzymatic Regulation Through Redox Modifications.

Redox-sensitive proteins are central to signaling pathways that regulate plant responses to environmental stress [32]. Resources like CPLM support large-scale proteomic studies by cataloging diverse post-translational modifications, aiding the analysis of protein modification networks [33]. For example, Balmant et al. [34] used tandem mass tag-based redox proteomics to identify proteins responsive to flg22 in guard cells, revealing redox-dependent regulation of photosynthesis, lipid binding, and defense signaling. In tomato, Wang et al. [35] used iodoTMT to identify 70 redox-sensitive peptides during fruit ripening. Oxidation of enzymes such as polygalacturonase 2A (PG2A) and 1-aminocyclopropane-1-carboxylate oxidase-like protein (E8) was linked to fruit softening (Figure 2).

**Figure 3:**
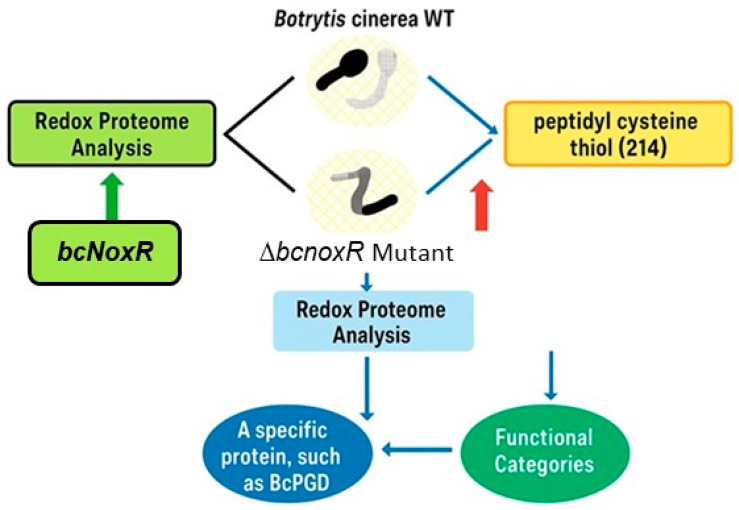
A proposed model of NADPH oxidase (NOX)-mediated redox regulation in *Botrytis cinerea*. This diagram illustrates a proposed model depicting NOX-mediated redox regulation in *Botrytis cinerea*, focusing on the deletion mutant ∆*bcnoxR*. The central element, ‘*bcnoxR*,’ is subjected to an iodoacetyl tandem mass tag-based redox proteomic assay, revealing protein redox dynamics. Comparison between the wild type and ∆*bcnoxR* mutant indicates 214 unique peptidyl cysteine (Cys) thiols from 168 proteins, with generally higher oxidation levels in ∆*bcnoxR* (indicated by a red arrow). Site-specific thiol oxidation analysis highlights 142 peptides significantly changing in abundance in ∆*bcnoxR*, linking to various functional categories. One key protein, 6-phosphate dehydrogenase (BcPGD), emerges as crucial for oxidative stress response and pathogenesis [37]. WT, wild-type. Arrows in the figure are color-coded to convey directional relationships: red arrows indicate increased thiol oxidation in the ∆*bcnoxR* mutant; the blue arrow represents a downstream connection from redox proteome findings to specific functional categories; the green arrow shows the methodological linkage from *bcNoxR* to redox proteome analysis. In plants, thioredoxins (Trxs) and glutaredoxins (Grxs) modulate redox states of metabolic enzymes. Trxs activate Calvin cycle enzymes, while Grxs regulate Fe-S cluster proteins for proper electron transport [38,39]. Redox modifications such as *S*-nitrosation and sulfenylation also control antioxidant enzymes like superoxide dismutase (SOD) and ascorbate peroxidase (APX) [40,41].

Redox-dependent modifications fine-tune enzymatic activity, impacting core metabolic pathways such as respiration and antioxidant defense. Redox proteomics has elucidated these regulatory mechanisms at the molecular level in model organisms like Saccharomyces cerevisiae [36]. For instance, in *Botrytis cinerea*, redox analysis revealed that deletion of *bcnoxR* gene altered thiol oxidation patterns in 214 peptides, including the key enzyme BcNoxR involved in NADPH production (Figure 3) [37].

### 2.3. Implications for Plant Development and Seed Germination

Redox signaling is fundamental to plant development. During seed germination, redox proteomics has uncovered dynamic shifts in cysteine oxidation regulated by Trx and GSH systems, which facilitate metabolic reactivation. Subsequent oxidative bursts mediated by RBOHs help establish redox homeostasis for seedling growth [32,42].

Floral development also involves redox regulation of gene expression patterns. GSH and its oxidized form (GSSG) are key players in cellular redox regulation, governing the redox potential and ensuring a stable environment within the cell. They are involved in the control of gene expression, impacting flower development [43]. Furthermore, redox-regulated proteins, such as ROXY1, are required to restrict the expression of key regulatory transcription factors like AGAMOUS (AG), which controls stamen and carpel organogenesis during flower development [44].

Building on the experimentally identified redox-sensitive proteins highlighted in Table 1, the following section shifts focus to computational prediction tools developed to identify and characterize oxiPTMs. While much of redox proteomics has historically relied on mass spectrometry and enrichment strategies, the integration of machine learning (ML) and deep learning (DL) approaches has enabled large-scale, proteome-wide inference of redox-sensitive cysteine residues. To help both computational and experimental researchers understand these tools, we summarize not only the types of predictions they make, but also the underlying algorithms, data sources, and performance metrics that guide their development and application in plant systems.

## 3. Computational Prediction Tools for Redox-Dependent PTMs

Computational tools have become essential for predicting redox-dependent post-translational modifications (oxiPTMs), especially when experimental validation is limited or resource-intensive. These tools are particularly useful for prioritizing candidate redox-sensitive proteins, designing site-directed mutagenesis experiments, or complementing mass spectrometry-based studies. Depending on the modification type and research goal, researchers can choose from tools based on sequence motifs, structural features, or redox proteomics data. In the following section, we summarize key predictive models and explain how they can be applied in redox biology, particularly in the context of plant stress responses.

Redox-dependent PTMs are chemical alterations in protein residues that occur in response to cellular redox fluctuations. Modifications such as *S*-glutathionylation and *S*-nitrosation, resulting from the attachment of GSH or nitric oxide (NO) to Cys residues, respectively, play a crucial role in modulating protein structure, function, and interactions. These oxiPTMs regulate key physiological processes in plants, including stress responses, metabolic pathways, and redox homeostasis. For example, *S*-nitrosation of SlTrxh downstream of SlMYB86 enhances nitrate stress tolerance in tomato seedlings [49]. However, their transient nature and low abundance present challenges for experimental identification.

Recent advancements in computational tools have significantly improved the prediction and characterization of oxiPTMs by integrating machine learning (ML), deep learning (DL), and statistical models. These tools analyze sequence features, structural properties, and network characteristics, enhancing the accuracy of identifying potential modification sites. Several models have been developed to predict *S*-nitrosation sites in plant proteins, aiding in the identification of redox-sensitive residues [17]. Additionally, studies have highlighted the role of cysteine residues in redox regulation, emphasizing the importance of oxiPTMs in plant metabolism and signal transduction [50].

**Figure 4:**
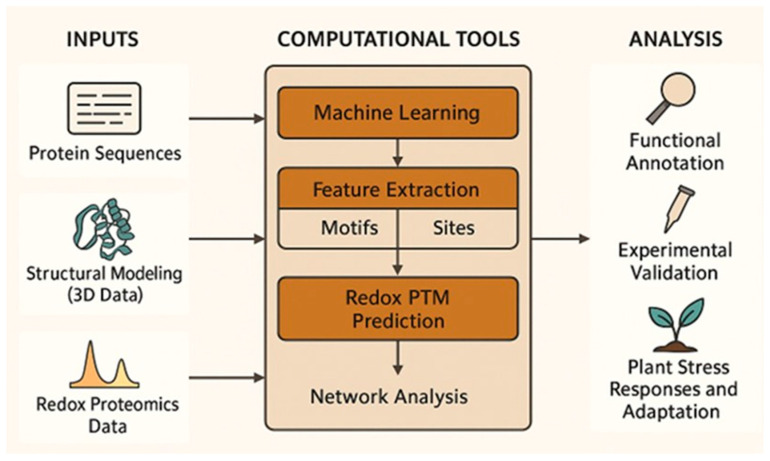
A computational framework for predicting redox-modified proteins. This figure illustrates a stepwise computational workflow for identifying and analyzing redox post-translational modifications (PTMs) in proteins. The process begins with three primary data sources: protein sequence analysis, structural modeling (3D data), and redox proteomics datasets. These inputs feed into computational tools that utilize machine learning algorithms, feature extraction (motifs, sites), and redox PTM prediction models to identify potential redox-sensitive sites. The predicted modifications undergo further functional annotation, validation with experimental approaches, and network-based redox analysis to integrate findings into broader biological pathways. The final goal is to enhance our understanding of redox regulation in plant stress responses and adaptation, facilitating advances in plant resilience and agricultural sustainability.

As shown in Figure 4, computational tools integrate sequence analysis, structural modeling, and machine learning to predict redox PTMs, which are validated through experimental and network-based approaches to understand redox regulation in plant stress responses.

### 3.1. Computational Approaches for Cysteine-Targeting Redox PTMs

Computational prediction of oxiPTMs leverages biologically relevant features, such as local sequence motifs around Cys residues, predicted structural properties (e.g., solvent accessibility, secondary structure), and evolutionary conservation, processed by machine learning (ML) or deep learning (DL) algorithms [51,52]. Tools such as those developed for redox PTM prediction employ advanced models, including support vector machines (SVMs), convolutional neural networks (CNNs), bidirectional long short-term memory networks (BiLSTMs), and attention mechanisms, to learn discriminative patterns from experimentally validated datasets [53,54]. Users typically input a protein sequence or proteome, and the models return predicted oxiPTM sites with associated confidence scores or functional annotations. Performance is commonly evaluated using metrics such as area under the ROC curve (AUC), Matthews correlation coefficient (MCC), and sensitivity/specificity [55,56].

The following subsections are organized based on the chemical nature and functional similarity of redox-related cysteine modifications.

#### 3.1.1. Gasotransmitter-Mediated Modifications

*S*-nitrosation and persulfidation are oxiPTMs mediated by gaseous signaling molecules such as NO and hydrogen sulfide (H_2_S), respectively.

##### *S*-Nitrosation

*S*-nitrosation denotes the covalent binding of a NO group to a Cys residue within a protein [57,58,59,60,61,62]. This dynamic and reversible process exerts regulatory control over the function, interaction, and localization of proteins [58,63]. Within plants, *S*-nitrosation plays pivotal roles in physiological processes like growth, development, stress response, and gene expression [61,64,65,66]. It is also related to plant immunity, development, and senescence [67]. Mass spectrometry-based redox proteomics has been widely used to detect and quantify *S*-nitrosation in biological systems [68,69]. These methodologies have revealed numerous *S*-nitrosated proteins and Cys in plants, highlighting the specificity of this PTM [66,67], while also advancing understanding of *S*-nitrosation in other organisms such as *Trypanosoma cruzi* [70]. Unraveling the molecular mechanisms and regulatory functions of NO signaling necessitates the identification of *S*-nitrosated proteins and their Cys residues [71,72].

More challenging is that the NO group in *S*-nitrosation is rather unstable, total modifications of this are very little, and other PTMs interfere with experimental techniques for detecting it [73,74]. A solution to this problem is the computational prediction of *S*-nitrosated Cys using sequence, structure, and/or network features [17,75].

Recent advancements in computational biology have greatly facilitated the prediction of *S*-nitrosated Cys residues, offering valuable alternatives to labor-intensive experimental methods. Early tools such as GPS-SNO v1.0 employed position-specific scoring matrices and machine learning trained on experimentally validated peptides, achieving 82% accuracy [76]. PreSNO, using a support vector machine integrated with random forest classifiers, attained 75% accuracy and an MCC of 0.252 [77], while RF-SNOPS implemented feature fusion strategies to reach 81.84% accuracy and an MCC of 0.814 [78]. More recent models have leveraged deep learning architectures. For instance, pLMSNOSite incorporates contextualized embeddings from protein language models and achieved MCC, sensitivity, and specificity scores of 0.340, 0.735, and 0.773, respectively, on independent datasets [79]. Among the most advanced predictors, SNO-DCA [80] integrates dense convolutional blocks, attention mechanisms, and one-hot encoded sequences, outperforming previous models, particularly in handling imbalanced datasets, as demonstrated by its superior MCC and AUC values. These tools collectively represent a growing suite of resources for high-confidence, proteome-scale *S*-nitrosation site prediction.

##### Persulfidation

Protein persulfidation is a reversible oxiPTM where a sulfur atom is added to the thiol group (-SH) of a Cys residue, forming a persulfide group (-SSH). This modification is primarily mediated by H_2_S and plays a significant role in redox signaling [81,82,83]. Recently, a substantial number of proteins susceptible to persulfidation have been identified using the dimedone-switch method combined with mass spectrometry [84]. This approach has been applied across various plant species and organs, including *Arabidopsis*, pepper, rice, and barley, under both physiological and stress conditions [84,85,86,87,88]. However, to our knowledge, no predictive programs have been developed to identify this oxiPTM.

#### 3.1.2. Redox Buffering-Dependent Modifications (GSH and ROS)

This group includes oxiPTMs such as *S*-glutathionylation, *S*-sulfenylation, sulfinylation, and general Cys oxidation. These modifications are closely linked to ROS metabolism and the GSH redox buffer system.

##### *S*-Glutathionylation

*S*-glutathionylation is a crucial redox-dependent PTM where GSH forms a disulfide bond with Cys residues, influencing antioxidant defense, apoptosis, metabolism, and signal transduction [89,90,91,92]. Identifying proteins and cysteines undergoing *S*-glutathionylation is essential for understanding its molecular basis and functional consequences [93,94]. However, experimental techniques for detecting *S*-glutathionylation suffer from limitations, including accuracy, selectivity, and diagnostic output capacity [95,96].

To address these challenges, computational methods have been developed to predict *S*-glutathionylated Cys using sequence features, structural characteristics, and network-based properties [33,97,98]. Anashkina et al. [90] introduced a novel approach employing a position-specific matrix developed from an independent dataset of over 200 heptapeptide sequences containing *S*-glutathionylated Cys. This method assigns a *S*-glutathionylation propensity score to each Cys and predicts modification sites based on predefined thresholds. Notably, it achieved 77% accuracy on a validation set of 140 experimentally verified *S*-glutathionylated proteins, demonstrating applicability even to proteins with unknown structures.

Zhao et al. [99] developed PGluS, a computational tool for predicting *S*-glutathionylation sites in proteins. This PTM, characterized by the formation of mixed disulfide bonds between Cys residues and GSH, plays a crucial role in redox regulation and cellular signaling. PGluS employs multiple sequence and structural features combined with support vector machines (SVM) to enhance prediction accuracy. It achieved an accuracy of 71% during five-fold cross-validation and 71% on an independent test dataset, demonstrating its potential applicability across various organisms, including plants.

Furthermore, Chen et al. [100] developed GSHSite, which utilizes an iterative statistical method to identify *S*-glutathionylation sites by analyzing sequence motifs and structural characteristics. This tool offers a web-based interface, allowing users to input protein sequences and receive predictions on potential *S*-glutathionylation sites, along with associated sequence motifs. By integrating computational predictions with experimental validations, these tools contribute to a better understanding of redox-based regulatory mechanisms in plant biology and other biological systems.

##### *S*-Sulfenylation and *S*-Sulfinylation

*S*-sulfenylation (–SOH) and *S*-sulfinylation (–SO_2_H) represent key oxidative modifications where Cys thiols are converted into sulfenic and sulfinic acids, respectively [101,102]. These modifications regulate protein function and redox homeostasis, mediating hydrogen peroxide signaling pathways [103]. However, *S*-sulfinylation is typically less reversible than *S*-sulfenylation and may indicate oxidative damage rather than regulatory signaling.

Zhang et al. [19] introduced BiGRUD-SA, a computational model for predicting protein *S*-sulfenylation sites, leveraging a bi-directional gated recurrent unit (BiGRU) and self-attention mechanism for enhanced accuracy. Using diverse feature extraction techniques, such as AAC, BLOSUM62, AAindex, EAAC, and GAAC, BiGRUD-SA achieved high accuracy (97%) on training datasets and 96% on independent test sets. Further validation on an *Arabidopsis thaliana* dataset confirmed its predictive robustness.

Similarly, Wang et al. [104] developed SulSite-GTB, an efficient predictor for cysteine *S*-sulfenylation sites that integrates amino acid composition and dipeptide composition features. Using the synthetic minority oversampling technique (SMOTE) for data balancing and LASSO for feature selection, SulSite-GTB achieved impressive accuracy (93%) and AUC values (0.9706), outperforming other state-of-the-art methods.

Lyu et al. [105] introduced LSTMWE, another powerful model for predicting cysteine *S*-sulfenylation sites. This predictor employs a long short-term memory (LSTM) network with word embedding and position embedding techniques. It demonstrated strong performance across different species, achieving an AUC between 0.82 and 0.85, an MCC of 0.838, and an AUC of 0.850 on human datasets.

Do et al. [106] developed fastSulf-DNN, a deep learning-based model designed for identifying *S*-sulfenylation sites. By integrating convolutional, recurrent, and attention layers, fastSulf-DNN delivered high predictive power, achieving an accuracy of 77%, sensitivity of 86%, specificity of 69%, MCC of 0.5554, and an AUC of 0.833 on independent test datasets.

Ning and Li [20] introduced DLF-Sul, a computational tool for predicting *S*-sulfinylation sites, which have historically been less studied. This model employs a multi-module deep learning architecture, integrating bidirectional long short-term memory (BiLSTM) networks and self-attention mechanisms while leveraging binary encoding, BLOSUM62, and amino acid index features. DLF-Sul achieved remarkable accuracy, with 92% sensitivity, 92% specificity, 92% overall accuracy, an MCC of 0.8416, and an outstanding AUC of 96%, making it a highly reliable predictor for researchers investigating this PTM.

Garrido-Bazán et al. [107] explored the structural implications of *S*-sulfinylation by using the PropKa server to assess the deprotonation potential of conserved DnmA Cys residues (C450 and C776). Theoretical pKa values indicated reduced deprotonation in aqueous environments, while pCysMod analysis identified C450 as a likely candidate for *S*-sulfinylation, with a false-positive rate of 0.56%.

##### Cysteine Oxidation

Cys oxidation is a crucial redox-dependent PTM in plants, involving the reversible modification of Cys residues by reactive species such as H_2_O_2_, NO, or H_2_S [2]. This process dynamically regulates protein activity, conformation, and interactions [108], impacting essential physiological functions, including signal transduction, enzyme catalysis, and antioxidant defense [109,110]. Accurately quantifying cysteine oxidation levels and protein abundances is essential for understanding redox-mediated protein regulation, but this remains challenging due to the low stoichiometry and diverse oxidation states of cysteine residues.

It is therefore essential to distinguish between tools that predict redox-sensitive Cys residues and those that quantify their oxidation status. Computational predictors (e.g., pCysMod, DLF-Sul) estimate the probability of modification at specific Cys sites using sequence or structural data, whereas tools such as CysQuant are designed to measure actual oxidation levels across conditions using isotopolog-labeled mass spectrometry. For instance, CysQuant enabled the quantification of Cys oxidation in *A. thaliana*, identifying disulfide-forming Cys in redox-sensitive enzymes under light stress [18]. This distinction highlights the complementary nature of predictive modeling and biochemical quantification in redox proteomics.

Several techniques have been developed for detecting and quantifying Cys oxidation, including mass spectrometry (MS), fluorescence labeling, and electrochemical detection [18,111]. These approaches have revealed the dynamic nature of Cys oxidation under varying environmental and developmental conditions in plants [13,94,112]. However, traditional methods often suffer from limitations in sensitivity, resolution, and applicability across different biological contexts.

Recent advancements in computational methods have significantly improved the quantification of Cys oxidation and protein abundance from MS data. One notable development in this field is CysQuant, introduced by Huang et al. [18]. CysQuant utilizes light/heavy iodoacetamide isotopologues for differential labeling of reduced and reversibly oxidized cysteines, analyzed through either data-dependent acquisition (DDA) or data-independent acquisition mass spectrometry (DIA-MS). This innovative approach enhances both sensitivity and coverage, offering a powerful tool for detailed assessments of Cys oxidation kinetics in complex biological systems. CysQuant enables the quantification of Cys oxidation levels and protein abundances, demonstrating broad applicability across different MS platforms and biological organisms.

Beyond MS-based quantification, computational tools have been developed to predict and analyze redox-sensitive PTMs. ConCysFind, introduced by Moore et al. [113], is a web-based tool designed to predict conserved Cys residues that may serve as redox PTM sites. This tool was validated through phylogenetic analysis and biochemical assays across 21 plant species, reinforcing its predictive accuracy. Additionally, DLF-Sul, developed by Ning and Li [20], is a deep learning framework for *S*-sulfinylation site prediction. It integrates bidirectional long short-term memory (BiLSTM), convolutional neural networks (CNNs), and multi-head self-attention mechanisms, achieving an impressive 92% accuracy and an AUC of 96%, further expanding computational tools available for redox biology research.

Another significant contribution to the field comes from Sanchez et al. [114], who classified protein Cys thiols into four reactive groups and developed the Balanced Oxidation Susceptible Cys Thiol Database (BALOSCTdb). This database comprises 161 reversibly oxidized thiols and 161 non-susceptible thiols, each characterized by twelve biochemical parameters. Using the C4.5/J48 decision tree classifier, cysteines were categorized into oxidation-susceptible and non-susceptible classes based on critical features such as distance to the nearest sulfur atom, solvent accessibility, and pKa values. The classifier correctly predicted 136 of 161 oxidation-susceptible thiols, achieving an 80% accuracy rate.

#### 3.1.3. Structural and Regulatory Thiol Modifications

Certain redox PTMs, such as reversible disulfide bond formation and generalized redox-sensitive Cys switching, influence protein structure, stability, and functional regulation. These modifications serve as molecular switches in redox signaling pathways and stress responses. This group also includes tools designed to predict multiple or overlapping cysteine modifications across different redox contexts.

##### Reversible Disulfide Bonds

Reversible disulfide bonds are pivotal redox-dependent PTMs that dynamically regulate protein structure, function, and interactions [115]. Formed between cysteine residues, these bonds can be reduced and reformed in response to cellular redox changes, playing a crucial role in maintaining protein homeostasis. Reduction agents such as glutathione facilitate their reversibility, ensuring adaptability in oxidative environments [116,117].

These dynamic disulfide linkages impact protein stability, folding, and enzymatic activity, engaging with key reductants such as thioredoxin and glutaredoxin. Their reversible nature is particularly important in signal transduction, cellular division, and stress responses, where they act as molecular switches regulating biological pathways [117,118]. Moreover, they play a regulatory role in proteins such as transcription factors and chaperones, ensuring proper cellular responses to environmental fluctuations. Their formation and reduction are tightly controlled by the cellular redox environment and enzymatic modification systems [9,10].

Despite the challenges of experimental methods in detecting and characterizing reversible disulfide bonds, computational tools have emerged as powerful alternatives. RevssPred, developed by Sun et al. [119], utilizes support vector machine (SVM) classifiers and multiple structural and sequence-based features to predict reversible disulfide bonds, achieving an accuracy of approximately 75%. This tool provides insights into structural determinants, revealing that shorter bond lengths and high cysteine content are key factors influencing bond reversibility.

Building on this, diSBPred, introduced by Mishra et al. [120], leverages machine learning models to predict cysteine bonding residues with high precision. The model demonstrated outstanding 10-fold cross-validation accuracies of 83% for individual Cys bonding and 94% for cysteine-pair bonding, outperforming the nearest neighbor algorithm by 7.39% in jackknife validation. Notably, diSBPred surpasses other models by 43.25% in balanced accuracy, making it a valuable tool for annotating cysteine bonding sites and enhancing experimental protein structure prediction (aiPSP) accuracy.

Another significant computational advancement is DiANNA 1.1, introduced by Ferrè and Clote [121]. This tool employs a spectrum kernel SVM to classify cysteines into reduced forms, half-cystines (disulfide-bonded cysteines), or metal-binding ligands (e.g., iron, zinc). As one of the earliest implementations of string-based kernel methods on sequence windows, DiANNA 1.1 represents a pioneering approach in computational cysteine classification and has significantly contributed to structural bioinformatics.

##### Multiple Cysteine Modifications

Cys residues undergo a diverse array of PTMs, playing a critical role in regulating protein function, signal transduction, and stress responses [122,123]. These modifications include *S*-sulfenylation, *S*-nitrosation, persulfidation, *S*-glutathionylation, and disulfide bond formation, each contributing uniquely to antioxidant defense and cellular regulation.

To facilitate the study of cysteine PTMs, Li et al. [16] developed pCysMod, a computational tool designed to predict multiple Cys modifications, including *S*-nitrosation and palmitoylation. Built on a deep neural network architecture incorporating convolutional and attention layers, pCysMod achieved AUC scores ranging from 0.793 to 0.876 on benchmark datasets, demonstrating strong predictive performance.

Additionally, Meng et al. [124] introduced CysModDB, a comprehensive database for cysteine PTMs. This platform stores 70,536 experimentally confirmed CysPTM sites and 21,654 modified proteins, offering functional and structural annotations. CysModDB integrates protein–protein interaction networks, provides gene enrichment analyses, and features visualization tools that map modified Cys residues onto protein structures. Its cross-references to external databases enhance comparative and integrative analysis of Cys modifications.

##### Redox-Sensitive Cysteines

Redox-sensitive cysteines serve as critical sensors and effectors in cellular redox homeostasis, responding dynamically to oxidative stress [22,125].

Hasan et al. [23] introduced IRC-Fuse, a computational predictor designed for redox-sensitive cysteines. Utilizing feature fusion techniques like concatenation, averaging, and stacking, IRC-Fuse integrates information from diverse sources, including sequence, structure, and physicochemical properties. The model demonstrated robust performance with an accuracy of 74% and an AUC of 0.807 on a dataset containing redox-sensitive cysteines. In comparative analyses, IRC-Fuse exhibited a significant improvement of 10% in accuracy and 13% in MCC over other existing methods when evaluated on independent test data.

To ensure the reliability of computational predictions, most redox PTM tools are benchmarked using independent test sets and evaluated with standard classification metrics such as area under the receiver operating characteristic curve (AUC), Matthews correlation coefficient (MCC), sensitivity, and specificity. These metrics provide a robust assessment of model performance, particularly in imbalanced datasets common to redox proteomics [126]. For instance, the BiGRUD-SA model, which predicts *S*-sulfenylation sites using a bidirectional gated recurrent unit and self-attention mechanism, achieved over 95% accuracy on independent test data and was further validated using *Arabidopsis thaliana* datasets [19]. Similarly, the CysQuant platform combines isotopolog-labeled mass spectrometry with data-independent acquisition (DIA-MS) to quantify cysteine oxidation levels in vivo, revealing biologically relevant redox-sensitive disulfides in Arabidopsis chloroplastic enzymes under light stress [18]. These examples underscore the importance of coupling computational predictions with experimental validation to confirm biological relevance and improve model generalizability.

Beyond residue-level prediction, several emerging tools adopt network-based approaches to infer how redox regulation propagates through cellular systems. These methods incorporate redox proteomics data with protein–protein interaction (PPI) networks, gene regulatory maps, or graph-based learning algorithms to identify functional redox hubs and signaling cascades. For example, recent studies have applied graph neural networks (GNNs) to learn topological patterns associated with redox-sensitive proteins in plant systems [127,128]. By leveraging these architectures, such models can reveal context-specific redox nodes and modification hotspots that may not be evident through sequence-based prediction alone.

Table 2 summarizes key computational tools developed for the prediction and analysis of redox-dependent post-translational modifications (PTMs) targeting cysteine residues. The listed tools encompass various machine learning and deep learning approaches, mass spectrometry-based quantification methods, and integrative databases, covering a wide range of PTM types, including *S*-nitrosation, *S*-glutathionylation, sulfenylation, sulfinylation, thiol oxidation, and disulfide bond formation. Additionally, comprehensive platforms like Plant PTM Viewer offer multi-PTM visualization and analysis capabilities specifically tailored for plant proteins.

Despite advances in computational redox biology, current prediction tools for thiol-based oxiPTMs face several limitations. Many are trained on data from model organisms like humans or *Arabidopsis*, reducing their applicability to other plant species. Their performance depends heavily on limited, often biased experimental datasets. Most tools lack integration of protein structural context or subcellular localization, factors that influence redox reactivity. Transient modifications like *S*-nitrosation and sulfenylation are especially prone to false positives. Moreover, standardized benchmarking across datasets is lacking. These challenges highlight the need for experimental validation and more diverse, high-quality datasets to improve prediction accuracy and relevance in plant systems.

Building upon these limitations, it is also crucial to emphasize that the prediction of redox-sensitive Cys residues, even when statistically robust, remains insufficient to establish biological relevance. In particular, confirming whether a predicted site functions as a thiol switch and modulates protein stability, activity, or interaction in planta requires direct wet-lab validation. This should include redox proteomics under physiological and stress conditions, as well as functional analyses using wild-type and site-directed Cys mutants. Without such in vivo studies, bioinformatic assertions alone cannot reliably demonstrate redox-dependent regulation.

## 4. Future Prospects

The field of redox proteomics is poised to make significant advancements, particularly with the integration of artificial intelligence (AI), machine learning (ML), and multi-omics technologies. These computational approaches will refine the identification and functional characterization of redox-sensitive proteins, leading to breakthroughs in plant stress resilience and metabolic regulation.

One of the most promising directions is the development of AI-driven predictive models for redox-sensitive cysteine residues and oxiPTMs. Machine learning algorithms, such as deep learning-based sequence analysis and structural modeling, can accurately predict redox-sensitive sites based on protein sequence features, solvent accessibility, and evolutionary conservation [16]. For example, convolutional neural networks (CNNs) and recurrent neural networks (RNNs) have been successfully applied to identify *S*-nitrosation and *S*-glutathionylation sites with high precision, improving upon traditional biochemical detection methods.

Another key advancement is the application of natural language processing (NLP)-based AI systems to automate redox proteomics data analysis. AI-powered tools can extract meaningful patterns from large proteomic datasets, reducing manual annotation errors and accelerating the discovery of novel redox-sensitive proteins. For instance, AI-driven mass spectrometry (MS) analysis platforms, such as DeepMS, can enhance peak identification and quantify redox modifications with higher sensitivity, facilitating large-scale redox proteomics studies.

Furthermore, the integration of AI with multi-omics approaches (transcriptomics, metabolomics, and lipidomics) will enable a holistic understanding of redox signaling networks. Advanced network-based machine learning models, such as graph neural networks (GNNs), can construct and analyze dynamic redox interaction maps, predicting how oxidative modifications influence cellular pathways and stress responses.

Beyond computational advancements, real-time redox sensing technologies, including AI-enhanced biosensors, are being developed to dynamically track redox changes in live cells. Smart biosensing systems using machine learning algorithms can interpret fluorescence or electrochemical signals in response to redox fluctuations, providing high-resolution insights into oxidative stress adaptation in plants.

As AI and machine learning techniques continue to evolve, they hold immense potential for revolutionizing redox proteomics, enabling faster, more accurate, and large-scale analyses of redox-sensitive proteins. These innovations will not only enhance our understanding of oxidative regulation but also contribute to precision agriculture, crop improvement, and stress resilience strategies in the face of climate change.

Additionally, there is a tool available called the Plant PTM Viewer 2.0 (https://www.psb.ugent.be/webtools/ptm-viewer/ accessed on 25 June 2025), which is a comprehensive, open-access platform designed to explore protein PTMs in plants [129,130]. It integrates data from over 62 profiling studies, covering more than 112,000 modified peptides and 14 diverse PTM types, including crotonylation and 2-hydroxyisobutyrylation, across multiple species such as Arabidopsis, tomato, soybean, and moss. The tool offers advanced features like a protein list analysis tool for PTM enrichment and re-analysis of large mass spectrometry datasets, enabling discovery of novel modifications. With a user-friendly interface for visualizing and querying PTM data, the Plant PTM Viewer supports hypothesis generation, target discovery, and systems-level insights into plant biology, making it a valuable resource for researchers investigating plant stress responses and regulatory mechanisms.

To provide a concise overview of emerging trends in the field, we summarize below several key future directions:

- Development of real-time redox sensors to monitor dynamic thiol modifications in living plant tissues.
- Integration of AI-enhanced biosensors for in vivo detection of redox PTMs under stress conditions.
- Application of deep learning and natural language processing to automate redox proteomics data analysis and annotation.
- Utilization of graph neural networks to model redox interaction networks and predict protein function within signaling cascades.
- Expansion of multi-species training datasets to improve the generalizability of redox PTM prediction tools across diverse plant systems.
- Advancement of single-cell redox proteomics to uncover cell-specific redox regulation and signaling heterogeneity.
- Integration of multi-omics platforms (transcriptomics, metabolomics, proteomics) to construct predictive redox regulatory networks for crop improvement.

## 5. Conclusions

Redox proteomics, coupled with computational modeling, has emerged as a powerful approach for unraveling the complex dynamics of redox-sensitive proteins in plants. By providing detailed insights into redox modifications and their functional consequences, this field has significantly contributed to our understanding of plant stress responses and metabolic regulation. The integration of multi-omics approaches, such as genomics, transcriptomics, metabolomics, and proteomics, has opened new avenues for exploring redox regulatory networks in plants. Computational tools and AI-driven predictive models have further enhanced our ability to identify and characterize redox-sensitive proteins with high accuracy. As technological advancements continue to drive progress in this field, redox proteomics will play a crucial role in developing stress-resilient crops and improving agricultural sustainability. Importantly, the integration of redox proteomics with AI and machine learning offers direct applications in real-world plant research, including predictive breeding programs aimed at enhancing stress tolerance, redox biomarker-based environmental monitoring, and rational design of crop varieties optimized for specific climates or soil conditions. While AI and machine learning tools offer powerful means for predicting redox-sensitive cysteine residues, it is essential to emphasize that these predictions must be experimentally validated through wet-lab approaches, such as mass spectrometry, site-directed mutagenesis, or thiol-switch assays. Only through such functional validation can the biological relevance of proposed redox modifications be confirmed.

## Figures and Tables

**Table 1:** Representative redox-sensitive proteins and proteomic strategies used to elucidate stress-responsive mechanisms in plants and fungi.

Protein(s)/Process	Plant/System	Stress or Trigger	Proteomic Method	Key Finding	Citation
PSI assembly factor PSA3 (Cys199/200 redox switch)	*Arabidopsis thaliana*	Fluctuating light	Label-free redox proteomics	Identified thiol switches regulating PSI stability	[6]
Lipid transfer protein LTP-II	*Brassica napus* guard cells	flg22 exposure (bacterial peptide)	cysTMTRAQ + TMT redox proteomics	LTP-II is redox-responsive and required for pathogen defense	[34]
Fruit ripening enzymes (e.g., E8, PG2A)	Tomato fruit	Ripening (ROS fluctuations)	IodoTMT-based redox proteomics	Identified 70 Cys-peptides from 51 proteins; Cys-307 in E8 pinpointed as redox-sensitive site	[35]
214 peptidyl cysteine thiols from 168 proteins	*Botrytis cinerea* (fungal pathogen)	Δ*bcnoxR* mutant (NOX regulatory subunit deletion)	IodoTMT-based redox proteomics	Mutant showed increased oxidation of 214 Cys sites; highlights NOXR’s role in redox homeostasis and pathogenesis	[37]
Mitochondrial thiol redox switches driving seed germination	*Arabidopsis thaliana* seeds	Seed imbibition (imbibition stage)	Targeted redox proteomics	Demonstrated redox kick-start of mitochondrial metabolism via thiol switches during early germination	[42]
Global cysteine redox landscape (~84 proteins)	*Arabidopsis thaliana*	H_2_O_2_ treatment	Isotope affinity labeling + MS	Mapped proteome-wide redox-responsive thiols in metabolism and signaling	[45]
967 total proteins; differential abundance linked to defense enzymes (endochitinases, peroxidases, GST, LTP)	*Sorghum bicolor* leaves	Infestation by *Chilo partellus*	Label-free quantitative proteomics	Resistant genotypes maintain photosynthesis and stress-response proteins; 68 proteins showed differential expression	[46]
Lipoxygenases (down-regulated), oleosins (up-regulated)	*Glycine max* (soybean pods)	Overexpression of *GmDGAT1-2*	Quantitative proteomics + lipidomics	436 DEPs and 180 DEMs identified; lipoxygenases down, oleosins up—linked to increased total oil and oleic acid	[47]
Chloroplast stress-responsive proteins (e.g., elongation factors, chaperones)	Plants (chloroplasts)	Oxidative stress	Thiol redox proteomics (review + MS)	Highlighted key redox-regulated circuits in chloroplast signaling and PSII repair	[48]

Abbreviations: DEMs, differentially expressed metabolites. DEPs, differentially expressed proteins. E8, protein involvea in regulating ethylene biosynthesis. flg22, flagellin 22. GmDGAT1-2, Glycine max acyl-CoA:diacylglycerol acyltransferase 1 and 2. GST, glutathione *S*-transferase. LTP, lipid transfer protein. LTP-II, lipid transfer protein II. MS, mass spectrometry. NOX, NADPH oxidase. NOXR, NOX regulatory subunit. PG2A, polygalacturonase 2A. PSA, Photosystem I Assembly. PSI, photosystem I. PSII, photosystem II. ROS, reactive oxygen species.

**Table 2:** Computational tools for predicting thiol-based oxidative post-translational modifications (oxiPTMs) targeting cysteine residues.

oxiPTM	Tool	Methodology	Performance	Web	Citations
Multiple Cys PTMs	pCysMod	Deep learning with sequence features, optimized via PSO	AUC: 0.793–0.876 across five PTMs	http://pcysmod.omicsbio.info (accessed on 25 June 2025)	[16]
Cysteine Oxidation	CysQuant	MS (DDA/DIA) with isotopologue labeling	Quantified avg. 18% cysteine oxidation in Arabidopsis	https://github.com/patrick-willems/CysQuant (accessed on 25 June 2025)	[18]
*S*-sulfenylation	BiGRUD-SA	BiGRU + self-attention	Acc: 95.91% (test)	Not specified	[19]
*S*-sulfinylation	DLF-Sul	Deep learning: BiLSTM + attention + CNN	Acc: 92.08%; MCC: 0.8416; AUC: 96.4%	https://github.com/ningq669/DLF-Sul (accessed on 25 June 2025)	[20]
*S*-Nitrosylation	GPS-SNO 1.0	Group-based prediction system (GPS)	Predicted 31,900 sites in Arabidopsis proteome	http://mapman.gabipd.org/web/guest/mapman (accessed on 25 June 2025)	[76]
*S*-Nitrosylation	RF-SNOPS	ML-based feature extraction and fusion	Accuracy: 81.84%	Specified but not accessible	[78]
*S*-Nitrosylation	pLMSNOSite	Protein language model (ProtT5), deep learning	Sensitivity: 0.735; specificity: 0.773	https://github.com/KCLabMTU/pLMSNOSite (accessed on 25 June 2025)	[79]
*S*-Nitrosylation	SNO-DCA	Deep learning (CNN, attention module)	Outperforms previous models	https://github.com/peanono/SNO-DCA (accessed on 25 June 2025)	[80]
*S*-glutathionylation	PGluS	SVM with multiple features	71.41% accuracy (train), 71.25% (test)	Specified but not accessible	[99]
*S*-glutathionylation	GSHSite	SVM using motifs and ASA	High performance; validated experimentally	Specified but not accessible	[100]
*S*-sulfenylation	SulSite-GTB	GTB + SMOTE + LASSO	Acc: 92.86% (train); 88.53% (test); AUC: 0.97/0.94	https://github.com/QUST-AIBBDRC/SulSite-GTB/ (accessed on 25 June 2025)	[104]
*S*-sulfenylation	DeepCSO	LSTM with word embedding	AUC: 0.82–0.85 across species	http://www.bioinfogo.org/DeepCSO (accessed on 25 June 2025)	[105]
*S*-sulfenylation	fastSulf-DNN	DNN using protein sequences as “biological language”	Acc: 77.09%; MCC: 0.5554; AUC: 0.833	https://github.com/khanhlee/fastSulf-DNN (accessed on 25 June 2025) Not specified	[106]
Conserved Redox PTMs	ConCysFind	Phylogenetic conservation analysis	Validated with redox proteins	https://bibiserv.cebitec.uni-bielefeld.de/concysfind (accessed on 25 June 2025)	[113]
Thiol Oxidation	COPA	Decision tree (J48) with 12 features	80.1% accuracy (LOO CV)	Not specified	[114]
Reversible Disulfides	RevssPred	SVM based on structural features	Acc: 75%; AUC: 0.751 (CV)	Specified but not accessible	[119]
Disulfide Bond	diSBPred	Stacked ML with sequence and structure features	Acc: 94.2% (cys-pair), 82.29% (cys-site), 43.25% over NNA	Specified but not accessible	[120]
Disulfide Connectivity	DiANNA	ANN (v1.0), SVM (v1.1) for oxidation state and disulfide connectivity	Not quantified	http://bioinformatics.bc.edu/clotelab/DiANNA/ (accessed on 25 June 2025)	[121]
Multiple Cys PTMs	CysModDB	Integrated database and tools for CysPTMs	Resource/tool integration	https://cysmoddb.bioinfogo.org/ (accessed on 25 June 2025)	[124]
Multiple PTMs (19 types in 2019; 33 types in 2024)	Plant PTM Viewer	Integrative plant PTM database; includes sequence overview, confidence scoring, BLAST (accessed on 25 June 2025) and search tools	~370,000 PTM sites (2019); +112,000 modified peptides in update (2024); includes 8 species total	http://www.psb.ugent.be/PlantPTMViewer (accessed on 25 June 2025)	[129,130]

Abbreviations: Cys, Cysteine. PTMs, Post-translational modifications. pCysMod, Deep-learning-based predictor of multiple cysteine PTMs. PSO, Particle Swarm Optimization, AUC, Area Under the Curve. CysQuant, Mass spectrometry-based cysteine oxidation quantification. MS, mass spectrometry. DDA/DIA, Data-dependent acquisition/Data-independent acquisition. BiGRUD-SA, Bidirectional Gated Recurrent Unit with Decay and Self-Attention. BiGRU, Bidirectional Gated Recurrent Unit), Acc (Accuracy), DLF-Sul (Deep Learning Framework for *S*-sulfinylation prediction), BiLSTM, Bidirectional Long Short-Term Memory), CNN (Convolutional Neural Network), MCC (Matthews Correlation Coefficient), GPS-SNO (Group-based Prediction System for *S*-nitrosation. RF-SNOPS, Random Forest-based *S*-nitrosation prediction system. ML, Machine Learning. pLMSNOSite, *S*-nitrosation predictor using Protein Language Model and deep learning. ProtT5, Protein Transformer T5: transformer-based protein language model. SNO-DCA, Deep Convolutional Attention-based *S*-nitrosation predictor. PGluS, Predictor of protein glutathionylation using SVM. SVM, Support Vector Machine. GSHSite, Glutathionylation site predictor using SVM and accessible surface area. ASA, Accessible Surface Area. SulSite-GTB, Sulfenylation predictor using Gradient Tree Boosting. GTB, Gradient Tree Boosting. SMOTE, Synthetic Minority Oversampling Technique. LASSO, Least Absolute Shrinkage and Selection Operator. DeepCSO, LSTM-based predictor for cysteine sulfenylation. LSTM, Long Short-Term Memory. DNN, Deep Neural Network. fastSulf-DNN, Deep-learning predictor using protein sequences as biological language. ConCysFind, Cysteine redox PTM predictor based on phylogenetic conservation. COPA, Cysteine Oxidation Prediction Algorithm using decision trees. LOO, Leave-One-Out cross-validation. CV, Cross-Validation. NNA, Nearest Neighbor Algorithm. diSBPred, Disulfide Bond Predictor using stacked ML. DiANNA, Disulfide Connectivity prediction using ANN and SVM. ANN, Artificial Neural Network. CysModDB, Database and predictor suite for cysteine PTMs. CysPTMs, Cysteine Post-Translational Modifications. BLAST, Basic Local Alignment Search Tool.
